# Assessing Parameter Suitability for the Strength Evaluation of Intramolecular Resonance Assisted Hydrogen Bonding in *o*-Carbonyl Hydroquinones

**DOI:** 10.3390/molecules24020280

**Published:** 2019-01-14

**Authors:** Maximiliano Martínez-Cifuentes, Matías Monroy-Cárdenas, Juan Pablo Millas-Vargas, Boris E. Weiss-López, Ramiro Araya-Maturana

**Affiliations:** 1 Programa Institucional de Fomento a la Investigación, Desarrollo e Innovación, Universidad Tecnológica Metropolitana, Ignacio Valdivieso 2409, Casilla 9845, Santiago 8940577, Chile; 2 Instituto de Química de Recursos Naturales, Universidad de Talca, Av. Lircay s/n, Casilla 747, Talca 3460000, Chile; maty.monroy@gmail.com (M.M.-C.); jpmillas@utalca.cl (J.P.M.-V.); 3 Departamento de Química, Facultad de Ciencias, Universidad de Chile, Las Palmeras 3425, Casilla 653, Santiago 7800003, Chile; bweiss@uchile.cl; 4 Programa de Investigación Asociativa en Cáncer Gástrico (PIA-CG), Universidad de Talca, Talca 3460000, Chile

**Keywords:** hydroquinone, polyphenol, hydrogen bond, DFT, NBO, QTAIM

## Abstract

Intramolecular hydrogen bond (IMHB) interactions have attracted considerable attention due to their central role in molecular structure, chemical reactivity, and interactions of biologically active molecules. Precise correlations of the strength of IMHB’s with experimental parameters are a key goal in order to model compounds for drug discovery. In this work, we carry out an experimental (NMR) and theoretical (DFT) study of the IMHB in a series of structurally similar *o*-carbonyl hydroquinones. Geometrical parameters, as well as Natural Bond Orbital (NBO) and Quantum Theory of Atoms in Molecules (QTAIM) parameters for IMHB were compared with experimental NMR data. Three DFT functionals were employed to calculated theoretical parameters: B3LYP, M06-2X, and ωB97XD. O^…^H distance is the most suitable geometrical parameter to distinguish among similar IMHBs. Second order stabilization energies ΔE_ij_^(2)^ from NBO analysis and hydrogen bond energy (E_HB_) obtained from QTAIM analysis also properly distinguishes the order in strength of the studied IMHB. ΔE_ij_^(2)^ from NBO give values for the IMHB below 30 kcal/mol, while E_HB_ from QTAIM analysis give values above 30 kcal/mol. In all cases, the calculated parameters using ωB97XD give the best correlations with experimental ^1^H-NMR chemical shifts for the IMHB, with R^2^ values around 0.89. Although the results show that these parameters correctly reflect the strength of the IMHB, when the weakest one is removed from the analysis, arguing experimental considerations, correlations improve significantly to values around 0.95 for R^2^.

## 1. Introduction

Hydrogen bonding (HB) is generally described as a stabilizing electrostatic interaction with a partly covalent character. Some of the most direct experimental evidence of the HB formation is the deshielding of the hydrogen atom involved in the interaction, which is easily observed by NMR spectroscopy [1,2]. It is well known that intramolecular hydrogen bonding (IMBH) has a key role in determining the structure and properties of biologically-active molecules, and their study in model compounds is relevant in drug design [3,4,5]. For example, IMBH formation is determinant for passive membrane permeability of cyclic peptides [6] and in lipophilicity [7]. 

Any intramolecular hydrogen bonding IMHB is governed by the equilibrium between closed (IMHB) and open (no IMHB) forms. Consequently, the implementation of IMHB considerations in drug discovery programs requires experimental methodologies to assess the position of these equilibria, which are dependent on the strength of each IMBH [8]. The ^1^H-NMR spectroscopy is the most common tool to measure the propensity of compounds to form IMHBs, nevertheless, up to now the throughput of NMR, and other traditional methods, is limited in obtaining structure–activity relationships (SAR) and driving rapid cycles of compound optimization [8]. However, current computational chemistry allows one to assess, theoretically, the strength of IMBH through parameters such as the distance hydrogen-acceptor, the distance donor-acceptor, and the hydrogen bond angle [9]. Among the theoretical estimated parameters for the HB interaction, those from Natural Bond Orbital (NBO) and Quantum Theory of Atoms in Molecules (QTAIM) analysis [9,10,11,12,13] are the most popular. In this field, DFT methodologies have proven to be valuable tools to calculate molecular properties with an appreciably low computational time demand compared with *ab initio* methodologies. 

Previously we have studied the properties of some *ortho*-carbonyl hydroquinones with capability to cross membranes and to reach the inner mitochondrial membrane. These compounds act either as oxidative phosphorylation (OXPHOS) uncouplers, complex I inhibitors, or exert both activities; as a consequence they exhibit antitumor [14,15,16] or antiplatelet effects [17], or both. Interestingly, small structural changes on the hydroquinone scaffold determine their biological activities [15]. All these molecules exhibit strong resonance assisted intramolecular hydrogen bonding, assessed by ^1^H-NMR spectrometry [18]. These compounds act either as weak acids into the mitochondrial membrane, or by losing a hydrogen atom (or alternatively losing an electron followed by deprotonation) to afford the corresponding semiquinone radical, which can interfere in cell signaling. The formation of IMHB plays a key role [19,20] on both properties. IMHBs have shown appreciable effects on the antioxidant and electrochemical properties of hydroquinones and related phenols [21,22]. 

On the other hand, the *o*-carbonyl hydroquinone motif is present on natural products with different biological activities, such as the anticancer drugs doxorubicin and daunorubicin [23], acylnaphtohydroquinones [24], shikonin [25], and in peyssonol A [26]. 

The precise assessment of the strength of the IMBH interaction has many difficulties. Determination of the H-bond energy is possible only for the intermolecular hydrogen bond: the energy of the intermolecular interaction is usually equal to the energy required to separate the two interacting molecules [1]. However, there is no clear definition of the hydrogen bond energy for the IMHB because the interacting entities cannot be fully separated [27].

Moreover, it is relatively easy to roughly calculate the relative strength of a series of IMBHs when they take place in molecules with large structural differences; however, to assess the relative strength of IMBHs in very similar molecules, establish correlations with experimental parameters, and use these data to make correlations with biological activities, requires an adequate choice of theoretical methodologies.

The aim of this work is to study the IMHBs experimentally and theoretically in a series of structurally similar *o*-carbonyl hydroquinones. Calculated NBO and QTAIM parameters of the IMHBs obtained by different DFT functionals (B3LYP, M06-2X, and ωB97XD) will be correlated with experimental values obtained by NMR. 

## 2. Results

**Scheme 1:**
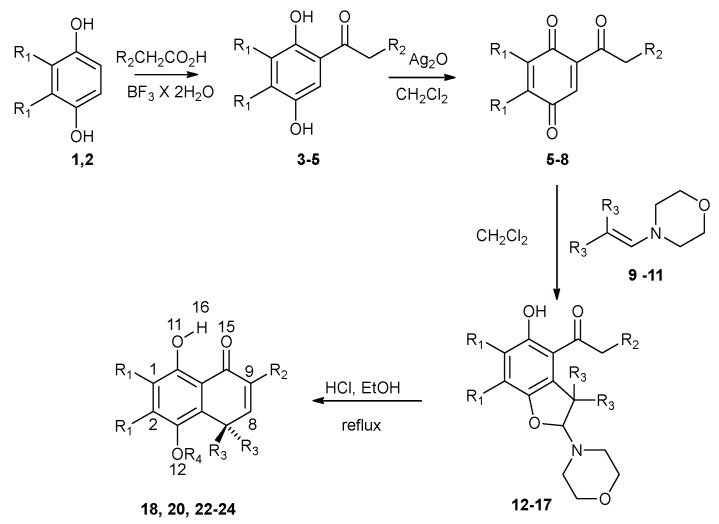
Synthesis of the bicyclic hydroquinones studied in this work.

The studied compounds were synthesized according to Scheme 1 [28,29]. Starting from hydroquinone (**1**) or 2,3-dimethylhydroquinone (**2**), the corresponding acylhydroquinones (**3**–**5**) were obtained by Fries rearrangement [30]. These compounds were oxidized with silver (I) oxide to obtain the corresponding quinones (**6**–**8**), which were not isolated. These quinones yield benzofurans (**12**–**17**) by reaction with the enamines (**9**–**11**). The last step involves an acid rearrangement of these benzofurans, yielding the corresponding bicyclic hydroquinones (**18**, **20**, **22**–**24**). Monomethyl ethers of hydroquinone **19** and **21** were obtained by methylation of hydroquinones **18** and **20**, respectively; they were synthesized to assess the effect of blocking the hydroxyl group not involved in IMHB.

Three hybrid functional were employed in this work: B3LYP, composed of Becke three parameters hybrid functional B3 and the non-local correlation functional of Lee, Yang, and Parr (LYP) [34,35,36]. The second corresponds to a hybrid meta-generalized gradient-approximation (hybrid meta-GGAs) functional, M06-2x, which have been described as one the of the best Truhlar group functionals for non-covalent interactions [37]. The last functional corresponds to the long-range corrected functional ωB97XD, which is designed to avoid rapid die-off of the non-coulomb part of the exchange functionals, which affects the modelling of long distance processes [38]. 

**Figure 1:**
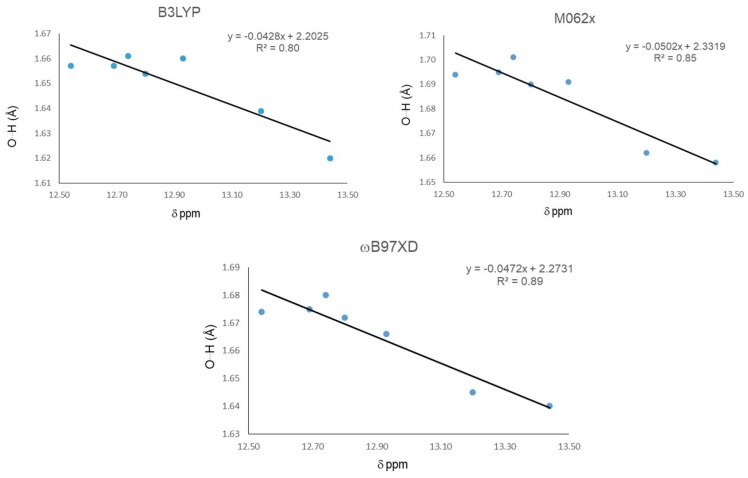
Correlations between H16^…^O15 distance and ^1^H-NMR chemical shifts (δ) for the hydrogen bond.

In this first part, we study main geometrical parameters from the IMHBs and their correlation with ^1^H-NMR chemical shifts. The results are presented in Table 1. Calculations were carried out at DFT level using the B3LYP, M06-2X, and ωB97XD functionals with the 6-311++g(d,p) basis set. Among the geometrical parameters, only the O15^…^H16 distances displayed enough differentiation to be used as comparison parameters for correlation with the chemical shifts of chelated protons. Compound **21** displayed the largest chemical shift and a more de-shielded proton, indicating the strongest IMHB, followed in strength by **20**. The later indicates that methyl substituents on C1 and C2 increase the IMHB strength. Additionally, the methylation at O12 and also a methyl group at C9 increase the strength of the IHB in **21** and **22**, respectively. The three functionals give the shortest O15^…^H16 distance for **21**, indicating the strongest IHB, which is in agreement with the largest downfield chemical shift. The smallest chemical shift for **18** indicates the weakest IHB in the series. Nevertheless none of the three functional gives the largest O15^…^H16 distance for **18**. These discrepancies can reflect the presence of intermolecular interactions competing with the IMHB, contributing in a non-negligible degree to the ^1^H-NMR spectra at the probe temperature, especially between pairs of molecules where one phenolic hydroxyl is replaced by a methoxy group. The same considerations can be applied to correlations with the next parameters. Global correlation among the chemical shift and O15^…^H16 distance for all molecules were carried out (Figure 1). Results indicate that the ωB97XD functional gives the best correlation with an R^2^ = 0.89, followed by M06-2X with a R^2^ = 0.85 and B3LYP, and the worst correlation with R^2^ = 0.80.

**Figure 2:**
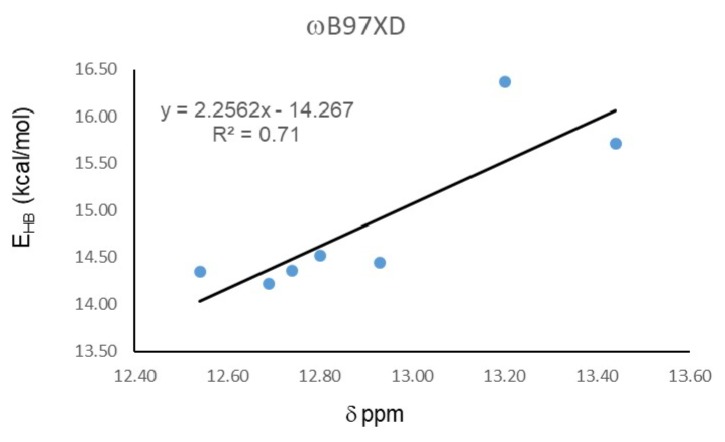
Correlation between Hydrogen bond energy (E_HB_) and ^1^H-NMR chemical shifts (δ).

In order to estimate the energy of IMHB, the open-close method was employed, which defines the H-bond energy as the difference between the open and close conformers [39,40]. Calculations were carried out with the functional ωB97XD, which presents the best correlation between experimental chemical shift and O16^…^H15 distance. Table 2 shows that energy for IMHB in the studied hydroquinones are around 15 kcal/mol. Hydroquinone **20** presents the highest value, 16.37 kcal/mol, while **19** presents the lowest IMHB energy, 14.22 kcal/mol. These energy differences are large enough to discard the contribution of the open form to the experimental NMR shieldings. Figure 2 shows the correlation between Hydrogen bond energy (E_HB_) and ^1^H-NMR chemical shifts (δ), which presents a R^2^ = 0.71, a value lower than the correlation with the O_15_^…^H_16_ distance (R^2^ = 0.89), calculated with the same functional. 

**Figure 3:**
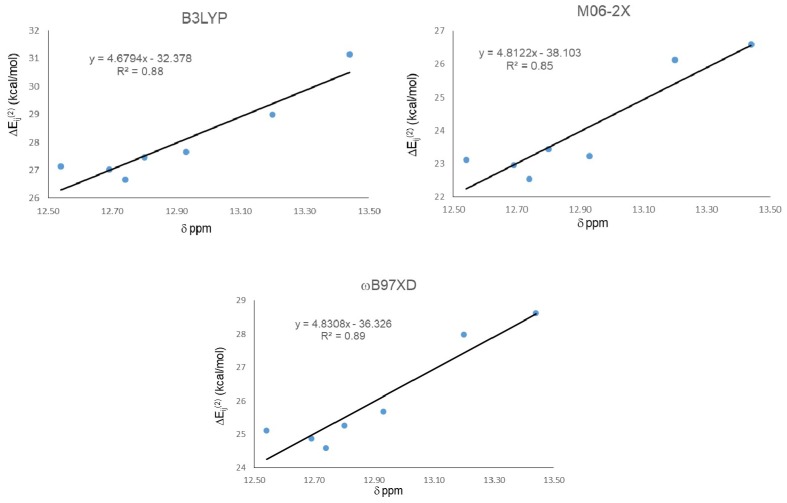
Correlations between second order stabilization energies ΔE_ij_^(2)^ LP O_15_ → σ* O_11_-H_16_ and ^1^H-NMR chemical shifts (δ) for IMHB.

The results from NBO analysis are presented in Table 3. Second order stabilization energies ΔE_ij_^(2)^ between donor (φ_i_) and acceptor orbital (φ_j_) allow one to study orbital interactions responsible for the IMHB. For all studied compounds, the HB donor atom is always an oxygen (two lone-pairs) and the acceptor is the antibonding sigma orbital of an O-H groups (σ^*^_OH_). As we found in the geometrical analysis, strong IMHB presence in **21** (the largest chemical shift) is in agreement with the largest ΔE_ij_^(2)^ obtained from the calculations with the three functionals. Nevertheless, ΔE_ij_^(2)^ did not predict well the weakest IMHB, corresponding to **18**, and the lowest stabilization energy was not predicted by any of the three calculations using the studied functionals. Global correlation among the chemical shift and ΔE_ij_^(2)^ for all HQs were carried out (Figure 3). As in the geometrical analysis, correlation results indicate that the best correlation is obtained with the ωB97XD functional, with R^2^ = 0.89. However, in this case it was followed by B3LYP with R^2^ = 0.87 and then M06-2X, the one that gave the worst correlation with R^2^ = 0.85. 

**Figure 4:**
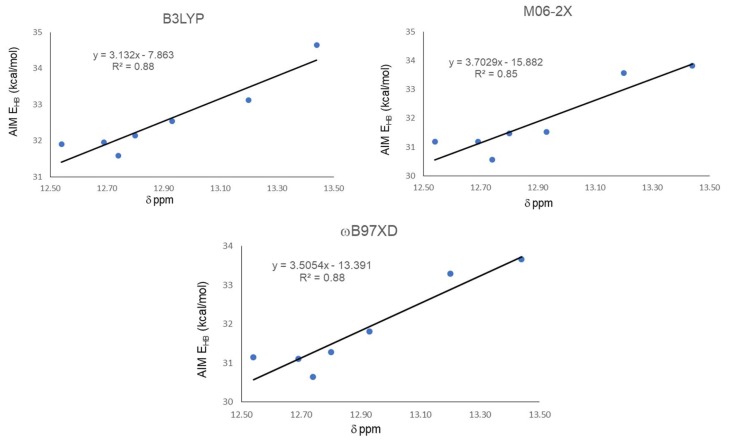
Correlations between hydrogen bond energy E_HB_ (kcal/mol) and experimental chemical shifts δ (ppm) of proton involved in IMHB.

The QTAIM analysis is presented in Table 4. It shows negative values for ∇^2^ρ and H for all studied compounds, indicating the covalent nature of their IHBs. |V|/G ratio is also a sensitive parameter for evaluating the covalency [41,42]. V can be interpreted as the pressure exerted on the electrons by the HB system, while G can be interpreted as the pressure exerted (as a reaction) by the electrons on the HB system, at the critical point. For negative values of ∇**^2^**ρ, as occurred in all our cases, values of |V|/G > 2 were indicative of covalent interaction [2]. Hydrogen bond energy (E_HB_) was obtained from the potential energy density (V) [13]. All values were slightly above 30 kcal/mol, while NBO analysis gave all energies slightly below 30 kcal/mol for the same interaction. These values were approximately twice those calculated by the open-close method, which indicates that NBO and AIM overestimate the interaction energy for the IMHB. E_HB_ values calculated by different methods have shown to be generally inconsistent [43]. As we found in both geometrical and NBO analysis, the strongest IHB is obtained for compound **21** and is well represented by QTAIM analysis for the three functionals, giving the highest E_HB_ for 21. On the other hand, similarly to NBO analysis, here QTAIM E_HB_ also did not adequately reflect the weakest IMHB present in **18**. Global correlations between QTAIM E_HB_ and chemical shifts are presented in Figure 4. A better performance is shown by ωB97XD and B3LYP with a R^2^ = 0.88. As in the above cases, M06-2X gave the worst correlation with R^2^ = 0.85.

The results shown in Figure 3 corroborates that correlations with the weakest IMHB of this series are not well represented by the computational calculations employed. In order to test what is the impact of the weakest IMHB on the correlations studied previously, compound **18** was eliminated from the data and correlations were obtained again. The results showed a significant improvement, without change in the relative position among different functionals and parameters studied. 

**Figure 5:**
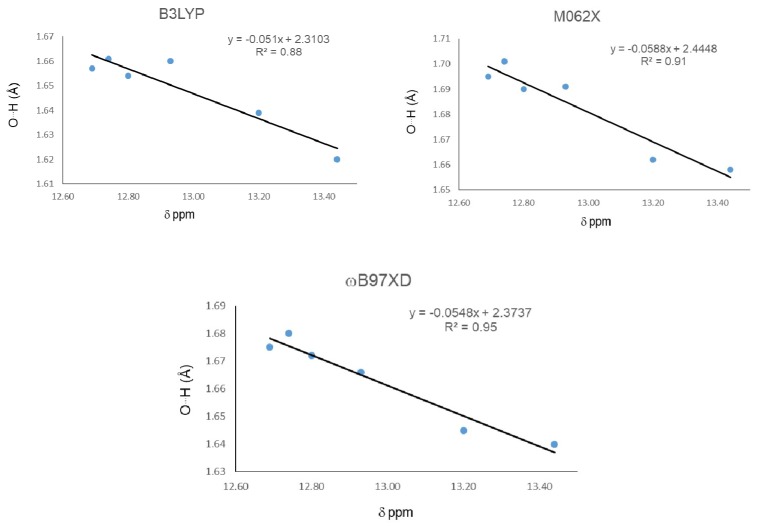
Correlations between O-H^…^O distance and ^1^H-NMR chemical shifts (δ) for the IMHB, excluding the weakest value.

Figure 5 shows correlations between ^1^H-NMR chemical shifts and O11-H16^…^O15 distance for the IHBs. R^2^ for B3LYP changed from 0.80 to 0.88, from 0.85 to 0.91 for M06-2x, and from 0.89 to 0.95 for ωB97XD. 

**Figure 6:**
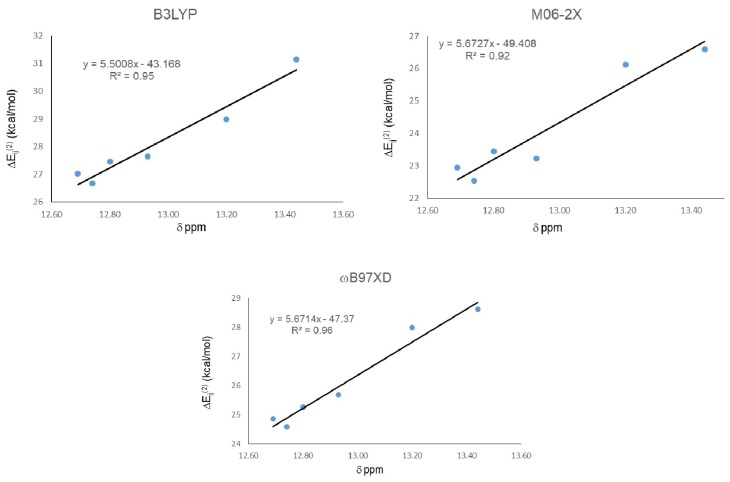
Correlations between second order stabilization energies ΔE_ij_^(2)^ LP O_15_ → σ* O_11_-H_16_ and experimental chemical shifts (δ) of protons involved in IMHB, excluding the weakest value.

Similarly, correlations between ^1^H-NMR chemical shifts and NBO energy for the IHBs are presented in Figure 6. R^2^ changed from 0.88 to 0.95 for B3LYP, from 0.85 to 0.92 for M06-2X, and from 0.89 to 0.96 for ωB97XD.

**Figure 7:**
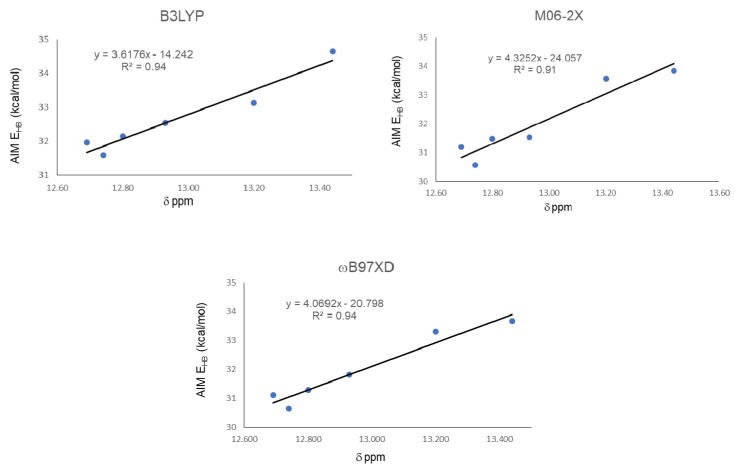
Correlations between hydrogen bond energy E_HB_ (kcal/mol) and experimental chemical shifts δ (ppm) of protons involved in IMHB, excluding the weakest value.

Finally, correlations between ^1^H-NMR chemical shifts and QTAIM energy for the IHBs, presented in Figure 7, showed a change in R^2^ from 0.88 to 0.94 for B3LYP, from 0.85 to 0.91 to M06-2X, and from 0.88 to 0.94 for ωB97XD.

## 3. Materials and Methods 

### 3.1. Synthetic Procedures

Melting points are uncorrected. All NMR spectra were acquired using a Bruker AVANCE DRX 300 spectrometer (Bruker BioSpin GmbH, Rheinstetten, Germany) operating at 300.13 MHz (^1^H) or 75.47 MHz (^13^C). Measurements were carried out at a probe temperature of 300 K.

#### 3.1.1. 1-(5-Hydroxy-3,3,6,7-tetramethyl-2-morpholino-2,3-dihydrobenzofuran-4-yl)ethan-1-one (**14**)

To a solution of 1-(2,5-dihydroxy-3,4-dimethylphenyl)ethan-1-one (400 mg, 2.2 mmol) in 20 mL of dichloromethane, Ag_2_O (1.28 g) was added and stirred with magnetic stirring for one hour. Then, the filtered solution was added over a solution of 4-(2-methylprop-1-en-1-yl)morpholine (314 mg, 2.2 mmol), in CH_2_Cl_2_ (10 mL), yielding 637 mg of the furan (**14**) (2.0 mmol, 90% yield). ^1^H-NMR δ(CDCl_3_): 1.37 (s, 3H, 3-CH_3_); 1.45 (s, 3H, 3-CH_3_); 2.14 (s, 3H, Ar-CH_3_); 2.19 (s, 3H, Ar-CH_3_); 2.33–2.60 (m, 4H, 2× CH_2_-O); 2.61 (s, 3H, CH_3_CO); 3.70 (m, 4H, 2× CH_2_-N); 4.68 (s, 1H, O-CH-N); 7.09 (s, 1H, 5-OH). ^13^C-NMR δ(CDCl_3_): 11.82; 12.51; 19.80; 31.17; 32.81; 45.07; 49.28; 66.75; 108.78; 120.94; 122.53; 123.74; 128.69; 147.01; 150.86; 205.43. M.P.: 140.1–142.2 °C. IR (KBr) ν/cm^−1^: 842.77; 1684.89; 2836.66; 2909.00; 2949.52; 2981.35; 3357.56. HRMS calculated: 319.1784; found: 319.1787.

#### 3.1.2. 5,8-Dihydroxy-4,4,6,7-tetramethylnaphthalen-1(4*H*)-one (**21**)

A solution of furan **14** (451 mg, 1.4 mmol) in ethanol (15 mL) was refluxed for 2 h, then the solution was poured in a mixture ice and water, and the solid precipitate was filtered and dried yielding hydroquinone 21 (256 mg, 78% yield). ^1^H-NMR δ(CDCl_3_): 1.61 (s, 6H, 2 × 4-CH_3_); 2.23 (s, 3H, Ar-CH_3_); 2.26 (s, 3H, Ar-CH_3_); 4.55 (s, 1H, 5-OH); 6.23 (d, 1H, *J* = 10 Hz, 2-H), 6.82 (d, 1H, *J* = 10 Hz, 3-H), 13.20 (s, 1H, 8-OH). ^13^C-NMR δ(CDCl_3_): 11.56; 13.04; 25.17; 38.03; 112.68; 123.43; 124.04; 131.51; 131.64; 143.52; 155.12; 160.73; 191.11. P.F.: 200.5–201.6 °C. IR (KBr) ν/cm^−1^: 1658.84; 2963; 2998.71; 3406.75. HRMS calculated: 232.1099; found: 232.1113.

#### 3.1.3. 8-Hydroxy-5-methoxy-4,4,6,7-tetramethylnaphthalen-1(4*H*)-one (**22**)

To a solution of hydroquinone **21** (100 mg, 0.43 mmol) in acetone (20 mL), powdered K_2_CO_3_ (85 mg, 0.6 mmol) and dimethyl sulfate (0.1 mL, 0.6 mmol) were added and stirred with magnetic stirring at reflux for 3 h. To the filtered mixture, a solution of KOH (5% in methanol) was added and kept at room temperature overnight. Then, it was neutralized with HCl 10% and extracted with CH_2_Cl_2_ 5 × 20 mL. The organic phase was washed with water and dried with anhydrous sodium sulfate, then was filtered and the solvent was eliminated at vacuum, by purification in flash column chromatography with hexane/ethyl acetate (5:1) as eluent, the product 22 was obtained (79 mg, 74% yield) ^1^H-NMR δ(CDCl_3_): 1.57 (s, 6H, 2 × 4-CH_3_); 2.20 (s, 3H, Ar-CH_3_); 2.31 (s, 3H, Ar-CH_3_); 3.72 (s, 3H, 8-OCH_3_); 6.24 (d, 1H, *J* = 10 Hz, 2-H); 6.78 (d, 1H, *J* = 10 Hz, 3-H); 13.44 (s, 1H, 5-OH). ^13^C-NMR δ(CDCl_3_): 11.39; 14.77; 27.09; 38.15; 61.49; 112.36; 124.13; 124.84; 137.88; 139.43; 149.29; 157.10; 160.76; 190.92. P.F.: 82.1–84.3 °C. IR (KBr) ν/cm^−1^: 1655.95; 2848.23; 2920.58; 2972.67; 3001.61; 3042.12; 3441.48. HRMS Calculated: 246.1256; found: 246.1265.

### 3.2. Computational Calculations

The calculations were carried out using the Gaussian 09 [44] program package (Revision a.01; Gaussian, Inc.: Wallingford, CT, USA). No symmetry constraints were imposed on the optimizations, which were performed at the DFT B3LYP/6-311++G (d,p) level. No imaginary vibrational frequencies were found at the optimized geometries, indicating that they are true minima of the potential energy surface. NBO analysis was performed using the NBOPro 6.0 [45] program package (NBO 6.0; University of Wisconsin: Madison, WI, USA). QTAIM analysis was performed using the AIM2000 [46] program package (Büro für innovative software, Bielefeld, Germany).

## 4. Conclusions

In this work, we found that O^…^H distance is the most suitable geometrical parameter for distinguishing among IMHBs of similar strengths. Second order stabilization energies ΔE_ij_^(2)^ from NBO analysis and hydrogen bond energy (E_HB_) obtained from QTAIM analysis were also able to properly distinguish among IMHBs in the studied molecule series. In all cases, ΔE_ij_^(2)^ from NBO give values for the IMHB lower than E_HB_ obtained from QTAIM analysis. ΔE_ij_^(2)^ are slightly below 30 kcal/mol, while E_HB_ from QTAIM gave values somewhat above 30 kcal/mol, which are approximately twice of those calculated by the open-close method. In all cases, parameters calculated using ωB97XD gave the best correlations with experimental ^1^H-NMR chemical shifts for the IMHBs, with values of R^2^ around 0.89. Despite this, the results showed that the employed parameters correctly described the strength of the IMHB, when the weakest one was removed from the analysis, correlations improved significantly to values around 0.95 for R^2^. These results can help to select the most suitable parameters for modeling molecules with IMHBs of similar strengths and achieving an adequate distinction between them through computational calculations. 

## Figures and Tables

**Table:** 

**Hydroquinone**	**R_1_**	**R_2_**	**R_3_**	**R_4_**
**18** [31]	H	H	Me	H
**19**^a^ [31]	H	H	Me	Me
**20**	Me	H	Me	H
**21** ^b^	Me	H	Me	Me
**22** [32]	H	Me	Me	H
**23** [32]	H	H	-CH_2_-(CH_2_)-CH_2_-	H
**24** [15,33]	H	H	Et	H

Note: ^a^ Obtained by methylation of **18**, ^b^ obtained by methylation of **20**.

**Table 1:** Experimental chemical shifts (δ) in ppm of protons involved in hydrogen bonding, and main geometric calculated parameters. Distances in angström, (Å), and angles in degree (°).

Molecule	δ ppm	Distance (O_15_^…^H_16_)			Distance (O_11_-H_16_)			Angle (O_15_^…^H_16_-O_11_)		
		B3LYP	M062x	ωB97XD	B3LYP	M062x	ωB97XD	B3LYP	M062x	ωB97XD
**18**	12.540	1.657	1.694	1.674	0.99	0.98	0.982	148	146	147
**19**	12.690	1.657	1.695	1.675	0.99	0.98	0.981	148	146	147
**20**	13.200	1.639	1.662	1.645	0.992	0.983	0.984	149	147	148
**21**	13.440	1.620	1.658	1.640	0.994	0.984	0.985	149	148	149
**22**	12.930	1.660	1.691	1.666	0.99	0.98	0.982	148	146	147
**23**	12.740	1.661	1.701	1.68	0.99	0.98	0.982	148	146	147
**24**	12.800	1.654	1.69	1.672	0.99	0.981	0.982	148	146	147

**Table 2:** Hydrogen bond energy (E_HB_), calculated as the difference between open and close conformers. Energies in kcal/mol.

Molecule	E_HB_
**18**	14.34
**19**	14.22
**20**	16.37
**21**	15.71
**22**	14.44
**23**	14.36
**24**	14.52

**Table 3:** Second order stabilization energies (ΔE_ij_^(2)^) between donor LP O_15_ and acceptor σ* O_11_-H_16_ orbitals involved in the IMHB. Energies in kcal/mol.

Molecule	ΔE_ij_^(2)^ LP O_15_ → σ* O_11_-H_16_		
	B3LYP	M06-2X	ωB97XD
**18**	27.14	23.12	25.11
**19**	27.02	22.95	24.87
**20**	28.99	26.13	27.99
**21**	31.15	26.6	28.62
**22**	27.65	23.23	25.68
**23**	26.67	22.54	24.59
**24**	27.47	23.44	25.27

**Table 4:** Atoms-in-molecule parameters for IHB of **18**–**24**. Electron density at the critical point ρ^BCP^ (a.u), its Laplacian ∇^2^ρ (a.u.), electron kinetic energy density G (a.u.), potential energy density V (a.u.), total electron energy density H (a.u.), and hydrogen bond energy E_HB_ (kcal/mol).

Functional	Molecule	ρ^BCP^	∇^2^ρ	G	V	|V|/G	H	E_HB_
B3LYP	**18**	0.0537	−0.0377	0.0459	−0.1013	2.2070	−0.1472	31.91
	**19**	0.0537	−0.0378	0.0460	−0.1015	2.2065	−0.1475	31.96
	**20**	0.0560	−0.0383	0.0478	−0.1051	2.1987	−0.1529	33.12
	**21**	0.0587	−0.0392	0.0501	−0.1100	2.1956	−0.1601	34.65
	**22**	0.0546	−0.0383	0.0469	−0.1033	2.2025	−0.1502	32.54
	**23**	0.0533	−0.0374	0.0455	−0.1003	2.2044	−0.1457	31.59
	**24**	0.0541	−0.0378	0.0463	−0.1020	2.2030	−0.1483	32.14
M06-2X	**18**	0.0454	−0.0408	0.0444	−0.0990	2.2297	−0.1434	31.19
	**19**	0.0454	−0.0409	0.0444	−0.0990	2.2297	−0.1434	31.19
	**20**	0.0490	−0.0427	0.0479	−0.1066	2.2255	−0.1545	33.57
	**21**	0.0495	−0.0428	0.0483	−0.1074	2.2236	−0.1557	33.83
	**22**	0.0458	−0.0412	0.0449	−0.1001	2.2294	−0.1450	31.53
	**23**	0.0446	−0.0402	0.0435	−0.0970	2.2299	−0.1405	30.57
	**24**	0.0459	−0.0410	0.0448	−0.0999	2.2299	−0.1448	31.48
ωB97XD	**18**	0.0510	−0.0380	0.0447	−0.0989	2.2125	−0.1435	31.14
	**19**	0.0508	−0.0381	0.0446	−0.0987	2.2130	−0.1434	31.10
	**20**	0.0546	−0.0394	0.0479	−0.1057	2.2067	−0.1536	33.30
	**21**	0.0553	−0.0396	0.0485	−0.1069	2.2041	−0.1553	33.66
	**22**	0.0518	−0.0386	0.0457	−0.1010	2.2100	−0.1466	31.81
	**23**	0.0502	−0.0376	0.0439	−0.0973	2.2164	−0.1412	30.64
	**24**	0.0512	−0.0381	0.0449	−0.0993	2.2116	−0.1442	31.29
